# Of ethically compromising positions and blatant lies about ‘truth serum’

**DOI:** 10.4103/0019-5545.31512

**Published:** 2007

**Authors:** A. K. Kala

**Affiliations:** Consultant Psychiatrist, 95-A, Model Gram, Ludhiana - 141002, India

When I was eleven years old, I went along with a friend to convey a message to his uncle who worked in a police station. In the courtyard, police were beating a hard-core pickpocket who already had enough beating marks on his body. A very old and thin looking policeman who was going off duty, stopped on seeing this, went to the senior in the beating team and requested with folded hands, “Huzoor, if you permit, can I also give him a slap?” Permission was given with a nod and the slap delivered.

I remembered the incident some months back while watching news on TV, which was showing narcoanalysis being performed on scamster Telgi. The man was lying down, semi-sedated, mumbling some words when prodded by light slaps on the cheeks by the psychologist who was holding a questionnaire. There were other people around, all in white coats. The TV clip itself went on for 20 minutes. The whole procedure must have lasted much longer. The mental health professional in the interrogating team reminded me of the old policeman. Both had no logical or scientific reason to do what they did. And it was certainly unethical to make it degrading by doing it on television. Remember, even if Saddam's death sentence was right, his death video was not and it shocked the conscience of the world. Sometimes, when you talk of ethics in such contexts, the question is asked, “Did he think of ethics while killing innocent people?” Answer to that is, that this is the difference between him and the civilized society.

Sir James Stephen stated in 1883, referring to a practice of police officers in India, “It is far pleasanter to sit comfortably in the shade rubbing red pepper into a poor devil's eyes than to go about in the sun hunting up evidence”.[1] Police have been plagued with a serious and long-standing image problem and have been constantly exhorted by everybody to be more scientific in their interrogation. They seem to have taken this too literally and have come up with this magical way to find truth, which, with its paraphernalia of syringes, drugs and white coats looks every bit “scientific”. In their over-enthusiasm, they forgot to verify reliability and scientific validity of the method, having been apparently overawed by its name ‘truth serum’. Had they enquired, they would have found that it often gives wrong and misleading results and on ethical grounds it is as slippery as, well, “rubbing red pepper into poor devil's eyes”. While police seem to have a reason to be doing it, why are mental health professionals, who should know better, conniving? The answer, I suspect is, narcissism.

Internationally, interest of security agencies in narcoanalysis is quite old, but not sustained. Scientific evidence against it as a method to reach truth has been consistent. It has had a somewhat better lease as a method to treat neurotic patients by exploiting their suggestibility and as a method of emotional catharsis. The suggestibility gets heightened under the effect of sodium pentothal but this can lead to false results when the method is used for detection of objective truth. While in neurotics, it is the perceived truth, which is important from patients' point of view even if it is objectively false, in a criminal investigation it can give wrong and confusing results. Even in neurotic patients, the method ceased to be used in the seventies because of lack of evidence that it was useful.

Inbau,[2] the then professor of law at Northwestern University who had had considerable experience in observing or participating in ‘truth serum’ tests, is of the opinion that such tests are occasionally effective on persons who, if they had been properly interrogated, would have disclosed the truth anyway. The person who is determined to lie will usually be able to continue the deception even under the effects of the drug. On the other hand, the person who is likely to confess will probably do so as the result of skillful police interrogation and it will not be necessary to use drugs.

The last good review on the subject entitled “Narcoanalysis and Criminal Law” was published in 1954 in American Journal of Psychiatry.[3] It concluded like this: “Criminal suspects, while under the influence of barbiturate drugs, may deliberately withhold information, persist in giving untruthful answers, or falsely confess to crimes they have not committed. Narcoanalysis is of doubtful value when used for the purpose of obtaining confessions to crimes. For ethical reasons the psychiatrist is advised against performing narcoanalysis when the examination is requested as an aid to criminal investigation”. Nothing positive has been published since then which favors narcoanalysis as a method of detecting truth.

In an infamous project called MKULTRA, the CIA promoted the use of LSD on unknowing subjects, which resulted in the death of one subject. The uproar and subsequent investigations in the 1970s, some of them by committees appointed by the US senate, confirmed these activities of the CIA. But they could not provide evidence against individuals because, in 1973, at the order of the then chief of the CIA, important records of the evidence were destroyed.[4]

After the attack on twin towers in September 2001 the security agencies revived their interest in unconventional methods of interrogation. The world was so petrified by the “ticking bomb” scenario that for sometime people closed their eyes to the niceties of ethical standards.

The turning point of narcoanalysis in India came in 2002. In June 2002, three months after the burning of a train bogie by a crowd at Godhra in Gujarat and the subsequent massacre of Muslims, seven persons accused of burning the train were brought to the Sree Sayaji General (SSG) Hospital in Vadodara. They were interrogated and doctors from the medical college department of anesthesia, surgery and psychiatry carried out a narcoanalysis. Since then it has been used very frequently in high profile criminal cases with a lot of pre and post test publicity in print and electronic media. In some cases, videos of the narcoanalysis actually being conducted were shown on television. Results of narcoanalysis however are not admitted as evidence in any country in the world, including India.

The Bombay High Court recently ruled that subjecting six of the accused in the multi-crore rupee fake stamp paper case to “certain physical tests involving minimal bodily harm” such as narcoanalysis, lie detector tests and brain mapping did not violate their constitutional rights, specifically the protection against self-incrimination guaranteed by Article 20(3). According to an editorial in The Hindu, this ruling opens the door to a systematic violation of human rights through the use of coercive pseudo-scientific practices masquerading as forensic methodology. It appears to place no restraint on the reprehensible enthusiasm of some investigative agencies for these methods. The judicial sanction for these methods of ‘lie-detection’ and ‘truth extraction’ rests on the argument that the protection of Article 20(3) does not apply at the investigative stage. The High Court ruling does not reflect the fact that these methods are deeply controversial worldwide - on scientific, legal and ethical grounds.

The editorial goes on, “The Bombay High Court ruling permits free polygraph testing as well as brain mapping of both accused and witness. Lie detectors or polygraphs, although physiologically benign, have consistently failed independent scientific scrutiny. Two comprehensive studies conducted in the United States (there are few takers for polygraphs across the Atlantic) in 1983 and 2002 found that polygraphy was marked by methodological chaos. Unlike genuine scientific disciplines, this field has made no progress in answering the serious scientific challenges before it. Polygraphy has no scientific theory worth the name behind it and there is little hope that one will emerge. But while the use of polygraphy is still current, no U.S. citizen can be compelled to take a polygraph test. Nor is polygraph evidence admissible in a court of law. Brain mapping for purposes of detecting lies is in an even more tentative state. It is backed by little forensic evidence or experience. The research literature on it is based mostly on laboratory studies under artificial circumstances. The Indian investigative agencies would do well to focus attention on the development of genuine multi-disciplinary forensic expertise rather than resort to coercive methods that are no more scientific than Pinocchio's nose”.[5]

It is interesting to note that other countries of the world do not share the enthusiasm of Indian police agencies about miraculous rediscovery of this technique of “scientific” interrogation. In fact most of the interrogation under narcoanalysis that is happening in the world today, happens in India. Thus India is not only the HIV capital of the world; it is also the “narcoanalysis” capital of the world! And most of this work happens in just two laboratories in the country. The team typically consists of psychologist, anesthetist and the representative of the security agency. A psychiatrist is often invited to join. Luis Justo[6] comes down heavily on such "biscuit" teams (behavioral science consultation teams).

In 1982, general assembly of United Nations adopted a resolution (number 37/194) which is entitled “Principles of Medical Ethics relevant to the Role of Health Personnel, particularly Physicians, in the Protection of Prisoners and Detainees against Torture and Other Cruel, Inhuman or Degrading Treatment or Punishment”. Principle 2 states that, “It is a gross contravention of medical ethics, as well as an offence under applicable international instruments, for health personnel, particularly physicians, to engage, actively or passively, in acts which constitute participation in, complicity in, incitement to or attempts to commit torture or other cruel, inhuman or degrading treatment or punishment”. Further, Principle 4(a) states that, “It is a contravention of medical ethics for health personnel, particularly physicians, to apply their knowledge and skills in order to assist in the interrogation of prisoners and detainees in a manner that may adversely affect the physical or mental health or condition of such prisoners or detainees and which is not in accordance with the relevant international instruments”.[7]

World Medical Association[8] recently revised its Tokyo declaration on this subject and now states inter-alia:

1. The physician shall not countenance, condone or participate in the practice of torture or other forms of cruel, inhuman or degrading procedures, whatever the offence of which the victim of such procedures is suspected, accused or guilty and whatever the victim's beliefs or motives and in all situations including armed conflict and civil strife.
2. The physician shall not provide any premises, instruments, substance or knowledge to facilitate the practice of torture or other forms or cruel, inhuman or degrading treatment or to diminish the ability of the victim to resist such treatment.

British Medical Association[9] published a report by a working group for this task. It was entitled, “Medicine Betrayed” and later published a handbook to serve as a guide for health professional working with security and police agencies.[10]

Justo[6] documents a recent statement of the American Medical Association: “Physicians must not conduct, directly participate in, or monitor an interrogation with an intent to intervene, because this undermines the physician's role as a healer.” The American Psychiatric Association has also reiterated its long-held position against the participation in or assistance to interrogation by psychiatrists.

Nearer home, Medical Council of India has effected a small but important addition to its official code of medical ethics, “The physician shall not aid or abet torture nor shall he be a party to either infliction of mental or physical trauma or concealment of torture inflicted by some other person or agency in clear violation of human rights”.[11] This code had been in force for more than two months when doctors administered sodium pentothal for an interrogation. But despite extensive media coverage of the doctor's participation in pharmacological torture, the medical council has not even sought an explanation, l *et al* one held an inquiry to demonstrate some resolve in implementing its own laws and ethical guidelines.[12]

In an incisive article in Indian Journal of Medical Ethics, Amar Jesani[12] has analyzed the ethical situation thus, "Three arguments were heard in the defense of these doctors. First, the use of sodium pentothal was not torture because it did not cause pain. Second, it was done in the national interest. And third, there is no harm in using torture if it can save lives. The first argument wrongly limits the definition of torture to pain. Torture includes the use of methods intended to obliterate the personality of the victim or diminish physical or mental capacities, even if they do not cause physical pain or mental anguish. Sodium pentothal tops the list of methods using ‘limited force’ advocated by organizations such as the US Central Intelligence Agency (CIA) whose human rights record needs no introduction. Interestingly, even the CIA acknowledges that it is advocating torture. Utilitarian arguments about national interest and saving lives are well known. This assertion is not backed by evidence that such interest was served, that what doctors did really saved lives. However, even if there is evidence of national interest, could it be used to justify acting unethically? The elevation of national interest above human morality has always had disastrous consequences. For the medical profession in India, the writing is on the wall. The BMA followed up Dr. John Dawson's assertion by producing a handbook on human rights for doctors, demonstrating its commitment to help doctors educate themselves and be ethical. The question is: Does the profession in India care?”

In a more recent article in the same journal, Jesani[4] further argues that, torture has made a renewed comeback in today's conflict-ridden world. However, sophisticated intelligence agencies know that torture is not only a violation of human rights; it also does not yield the desired results. A person who is being tortured usually admits to any crime attributed to him or her and gives information that the torturer would like to hear.

Heath professional in general are not known to be particularly media savvy. Glare of the camera makes us nervous and sometimes we want to show off. A couple of years back, a young girl was raped by a group of boys in Ahmedabad on new year night. She reported it to police and was medically examined. Two days later, she committed suicide. The lady doctor who had conducted her medical examination had no hesitation in talking about it on TV. “Oh, poor girl, she was in such a bad shape!!”

And a couple of months back, a psychiatrist who had been part of a “biscuit team” had no inhibition at all to talk about the details of narco-analysis session on TV. He was in fact quite effusive.

And that brings me back to my original questions, Why are mental health professionals being such willing partners to something which is neither scientific nor ethical?

The answer I do think, is plain and simple narcissism. You get to wear white coats on TV and save the nation from serial killers at the same time.
